# Appetite Sensations, Appetite Signaling Proteins, and Glucose in Obese Adolescents with Subclinical Binge Eating Disorder

**DOI:** 10.1155/2014/312826

**Published:** 2014-03-11

**Authors:** Kristi B. Adamo, Shanna L. Wilson, Zachary M. Ferraro, Stasia Hadjiyannakis, Éric Doucet, Gary S. Goldfield

**Affiliations:** ^1^Healthy Active Living and Obesity Research Group, Children's Hospital of Eastern Ontario Research Institute, 401 Smyth Road, Ottawa, ON, Canada K1H 8L1; ^2^School of Human Kinetics, Faculty of Health Sciences, University of Ottawa, Ottawa, ON, Canada K1N 6N5; ^3^Department of Pediatrics, Faculty of Medicine, University of Ottawa, Ottawa, ON, Canada K1N 6N5; ^4^Division of Endocrinology, Children's Hospital of Eastern Ontario, Ottawa, ON, Canada K1H 8L1; ^5^School of Psychology, Faculty of Social Sciences, University of Ottawa, Ottawa, ON, Canada K1N 6N5

## Abstract

*Objective*. This study aimed to investigate potential differences in appetite sensations, ghrelin, peptide YY, and glucose and their relationship with energy and macronutrient intake in obese adolescents with subclinical binge eating disorder. *Methods*. Fifteen obese adolescents (six and nine individuals with and without subclinical binge eating disorder, resp.) qualified for this study. Visual analog scales and Three-Factor Eating Questionnaires were used to assess eating behaviours. Circulating ghrelin, peptide YY, and glucose were measured after fasting and at multiple time points postprandially following a standardized breakfast meal. Energy and macronutrient intake were measured with an *ad libitum* lunch buffet. *Results*. Emotional eating scores were significantly higher in obese adolescents with subclinical binge eating disorder. Hunger levels rose and satiety levels fell significantly over the course of the monitoring period but there was no difference between the two groups. Obese adolescents with subclinical binge eating disorder did not have significantly different levels of appetite signaling proteins or glucose. Obese adolescents with subclinical binge eating disorder had a nonsignificantly higher energy and macronutrient intake. *Conclusions*. A significant difference between the two groups in terms of their emotional eating scores highlights the important role that psychological factors play in relation to eating behaviours.

## 1. Introduction

Health complications associated with obesity are a major public health concern, especially in the adolescent population who may carry adipose-related disease risk (i.e., type 2 diabetes, cardiovascular disease) forward into adulthood [1]. Of particular interest in adolescents is the presence and severity of eating disorders and the potential associations with obesity. Binge eating disorder (BED) is a psychiatric (eating) disorder characterized by the rapid intake of large quantities of food in a short period of time, relative to what most people eat in a similar time frame [2]. There is also an associated sense of loss of control (LOC), a lack of compensatory behavior characteristic of bulimia nervosa, and the presence of clinically significant distress [2]. This disorder affects 1–5% of adults [3], and 2% of children and adolescents [4]. At least 30% of obese children presenting to obesity management clinics have subclinical binge eating disorder or full-fledged BED [5], which contribute to obesity onset and maintenance [6]. However, it can often be difficult to diagnose individuals with binge eating disorder due to the stringent criteria and ambiguous notion of what constitutes a large amount of food. In a study of 105 children (6–13 years of age) with no full-fledged BED diagnosis, 30% of study participants noted the presence of one LOC episode during their lifetime [7]. Individuals with subclinical binge eating disorder (BE) do not present with all criteria required for a full BED diagnosis and thus it is pertinent to study these adolescents in regard to appetite regulation and control of food intake [8].

Hunger and satiety cues are paramount in regulating food intake in the general population but are also especially critical for individuals with BE who experience LOC over their eating. In a study of obese women with and without BED, there was a significant difference in satiety levels at the end of a meal but no change in hunger or satiety over the meal duration nor was there a difference in hunger and satiety ratings after adjusting for the amount of food consumed [9]. Mirch et al. found no significant differences in hunger and satiety in overweight children prior to access to an* ad libitum* lunch following a standardized breakfast [10]. However, they identified that the children with BE were satiated for a shorter time period following the standardized breakfast and had a higher energy intake at the buffet [10].

Ghrelin, a hunger-signaling protein, is low in adult obesity [11], and, similarly, obese children have significantly lower levels than those with normal weight [12]. Monteleone et al. found no difference in ghrelin levels between the two groups of obese women (with and without BED) at a single fasting time point [13]. However, Geliebter et al. found that obese women with BED, as compared to nonbinge eating controls, had lower levels of fasting ghrelin and significantly lower concentrations over time [14].

Inconsistent evidence to date on the effects of obesity on peptide YY (PYY), a satiety-signaling protein, underscores the need for further investigation in both adult and adolescent populations [15–18]. In regard to binge eating, Geliebter et al. found no differences in PYY levels in a study of obese women with and without BED following a liquid mixed meal [19].

The glucostatic theory of food control postulates that decreases in circulating glucose signals hunger as well as the initiation of the next meal and this theory has received partial support from animal models as well as human trials [20, 21]. It has been further confirmed that satiety is related to rapid changes in blood glucose and ghrelin concentrations [22]. However, glucose concentrations were not significantly different between obese women separated into three different groups (nonbinge eating control, subclinical BED, and BED) when measured at a single time point or over time [14].

The aim of this study was to determine potential differences between adolescents with and without BE by investigating appetite sensations as well as fasting and postprandial responses of appetite signaling proteins and glucose. Our secondary outcome was to determine the relationship between these factors and energy and macronutrient intake in obese adolescents with BE. We hypothesized that obese adolescents with BE would report less postprandial satiety and greater hunger following the standardized breakfast. We also hypothesized that obese adolescents with BE would have a lower suppression of ghrelin following a standardized breakfast and a smaller increase in PYY. Finally, we hypothesized that appetite sensations, appetite signaling proteins, and glucose may be related to increased energy and macronutrient intake in obese adolescents with BE during an* ad libitum *lunch buffet, either individually or as a combination of the different factors.

## 2. Materials and Methods

### 2.1. Participants

Participants were recruited specifically for this study through the pediatric endocrinology clinic at the Children's Hospital of Eastern Ontario (CHEO). Approval was obtained from the CHEO Research Ethics Board prior to study initiation with informed assent/consent was obtained from the adolescents and their parents prior to participation. Body weight was assessed using a balance beam scale and height was assessed using a SECA stadiometer. Obesity was defined as having a BMI ≥ 95th percentile based on age and sex [23]. Body composition was measured using foot-to-foot bioelectrical impedance analysis using the Tanita TBF-310. Waist measurements were taken according to standard measurement procedures. The females included in this study were postmenarchal and the males were postpubertal according to Tanner Stage 4 and serum testosterone levels. Participants were excluded if they had type 2 diabetes or were taking medications and/or supplements (e.g., metformin) that could have an effect on body composition or appetite. Participants were classified as having BE if they self-reported binge eating at least once per week in the last three months as assessed by the Eating Disorder Diagnostic Scale, such that this group could be considered to have a subclinical form of BED [24]. Control participants had no history of regular binge eating episodes (<5 episodes in their lifetime and none in the past 3 months).

### 2.2. Protocol

Participants arrived at the laboratory following an overnight fast and a blood sample was drawn. Participants were then provided with a 571 kcal standardized breakfast with a fixed macronutrient content (50% CHO, 35% FAT, and 15% PRO) and were instructed to consume the meal within 20 minutes. Blood measurements were taken postprandially at 15, 30, 60, 90, 120, and 240 minutes. At the end of four-hour monitoring period, participants were asked to complete an 18-item Three-Factor Eating Questionnaire (TFEQ-18), which consists of three categories of eating behaviours (cognitive restraint, uncontrolled eating, and emotional eating) [25] and is a modified version of the original TFEQ questionnaire [26]. Immediately following the last blood draw, participants were provided with an* ad libitum* preweighted buffet lunch, in privacy, and instructed to eat as much as they wanted in 45 minutes. Hunger and satiety were assessed using 150 mm visual analog scales (VAS) at fasting, at blood sampling time points, and immediately after the* ad libitum* lunch buffet [27]. Energy and macronutrient intake were measured based on the remaining food from the buffet and was analyzed using the ESHA Food Processor SQL dietary analyses software (ESHA Research, USA), using the 2007 Canadian Nutrient File.

### 2.3. Assays

Blood samples were collected in vacutainers containing EDTA and protease inhibitor aprotinin. The protease inhibitor dipeptidyl peptidase-IV (DPP-IV) was also added to the total PYY sample. All of the samples were kept on ice and centrifuged within 30 minutes of collection. Total PYY was analyzed using ELISA (Millipore, USA, Cat number EZHPPYT66K; Intra-assay CV [16.7%]; Interassay CV [22.1%]). Total ghrelin was also measured using ELISA (Millipore, USA, Cat number EZGRT-89K; Intra-assay CV [15.1%]; Interassay CV [17.5%]). All ELISA samples were done in duplicate. Glucose was measured using the Cholestech LDX system (Alere, USA).

### 2.4. Statistical Analysis

Analysis of covariance (ANCOVA) and chi-squared tests (categorical variables) were used to analyze potential differences in participant characteristics, baseline appetite sensations and appetite signaling proteins, and buffet lunch characteristics. Four-hour area under the curve (AUC), using the trapezoidal method, was calculated for the temporal hunger and satiety VAS responses (range = 15 to 240 minutes), interpolating for any missing questionnaire time points [28]. Linear mixed models (LMM) were utilized to assess potential longitudinal differences in appetite sensations, appetite signaling proteins, and glucose. These models defined group as the independent factor with time as the repeated measure and utilized a first-order autoregressive (AR1) covariance matrix. Partial correlations were calculated at the end of the monitoring period between appetite sensations and energy/macronutrient intake with appetite signaling proteins, glucose, and TFEQ factors. All performed correlations were assessed using a significance threshold defined by a Bonferroni-adjusted *P* value. All analyses were adjusted for sex and fat mass (kg). Results are presented as mean ± standard error mean (SEM). Significance was set at a threshold value of *P* < 0.05, unless otherwise stated. All analyses were performed using SPSS 20.0 (IBM, USA).

## 3. Results

In total, 23 participants consented to participate in the study. However, four participants were unable to complete the study because of difficulties with the blood draw procedures, fainting, or other illness. Additionally, one participant was deemed ineligible after the fact due to an error in BMI calculation and three participants were excluded due to medication changes between recruitment and assessment. Overall, 15 adolescents from 11 to 17 years of age participated in this study with 6 individuals classified as having BE and 9 in the control group without BE.

**Figure 1:**
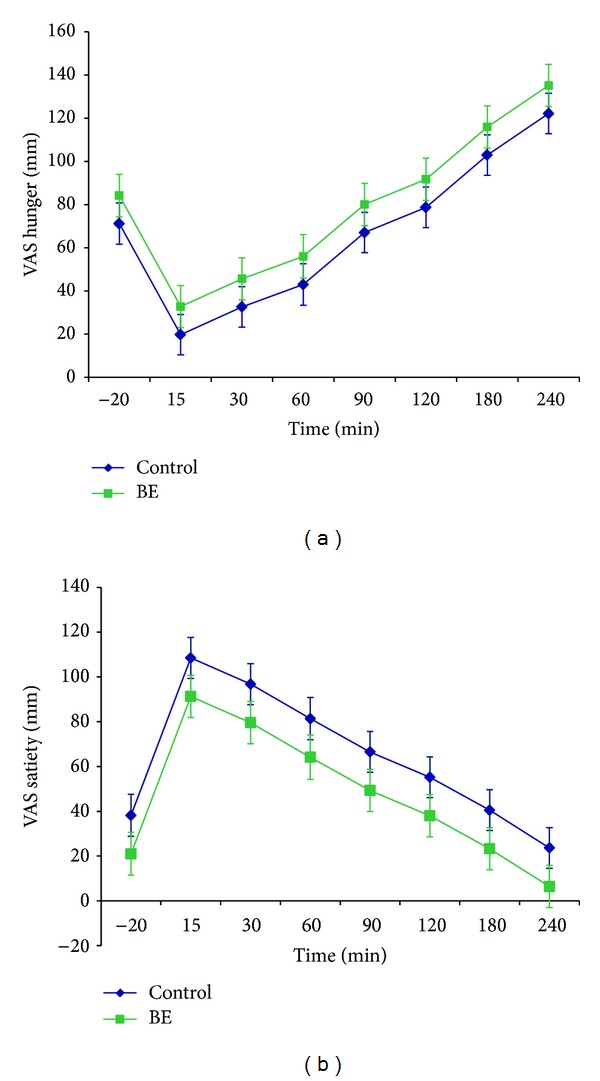
VAS hunger and satiety in obese adolescents with and without BE. Temporal changes in hunger and satiety during a four-hour monitoring period following a standardized, mixed breakfast. Error bars represent ±1 SEM. VAS: visual analog scale; BE: subclinical binge eating disorder.

**Figure 2:**
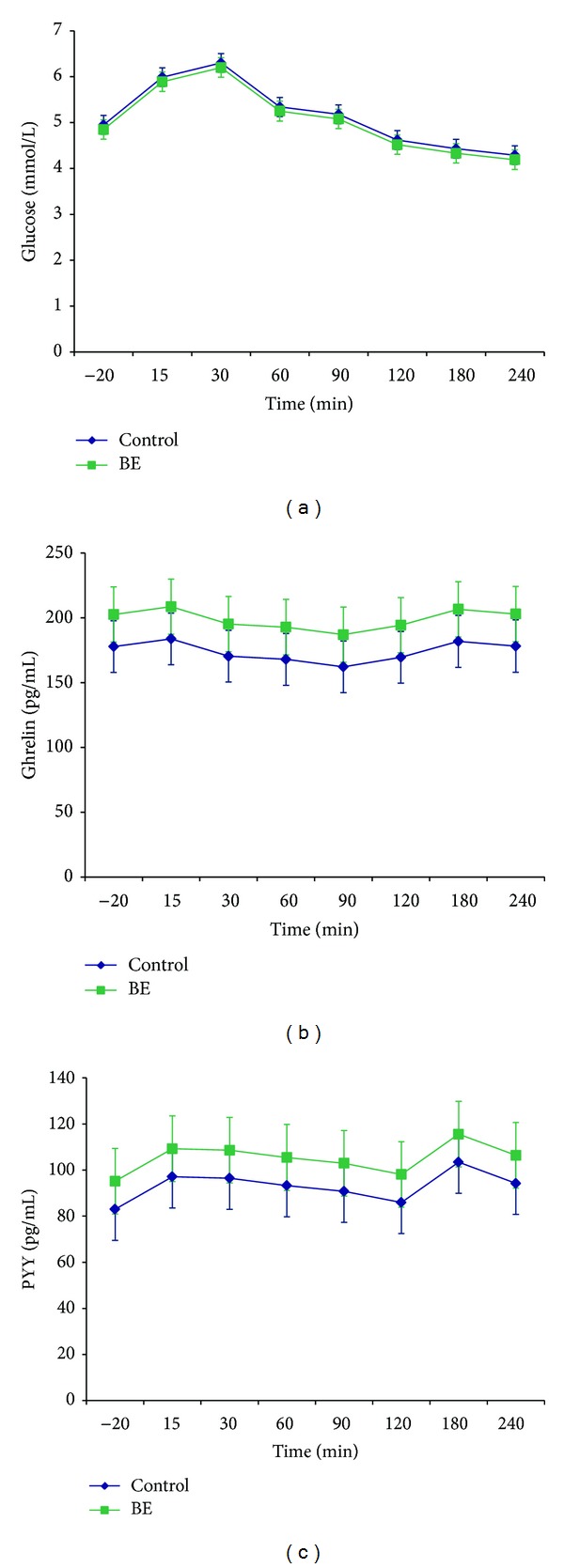
Glucose, ghrelin, and PYY in obese adolescents with and without BE. Temporal changes in glucose, ghrelin, and PYY during a four-hour monitoring period following a standardized, mixed breakfast. Error bars represent ±1 SEM. PYY: peptide YY; BE: subclinical binge eating disorder.

Participant characteristics, including demographics and baseline measures of appetite sensations, appetite signaling proteins, and glucose, were not significantly different between obese adolescents with and without BE (Table 1). A significant rise in hunger (*P* < 0.001) and decrease in satiety (*P* < 0.001) occurred after the standardized breakfast (Figure 1). Obese adolescents with BE had nonsignificant lower overall satiety scores (47 ± 7 versus 63 ± 6, *P* = 0.07) using the LMM, and there was no difference in hunger scores (Figure 1, Table 2) using the LMM or the 4 hr AUC. Glucose levels slowly decreased after the standardized breakfast (*P* < 0.001) but there was no change in ghrelin or PYY concentrations following the meal. Overall concentrations of glucose, ghrelin, and PYY across the monitoring period did not significantly differ between the two groups (Figure 2).

As shown in Table 3, obese adolescents with BE had higher emotional eating scores (TFEQ-18) as compared to the control group (9.09 ± 0.65 versus 5.07 ± 0.60; *P* = 0.002). While higher cognitive restraint and uncontrolled eating score were present in obese adolescents with BE, they did not significantly differ between the groups. 

As for energy intake at the* ad libitum* lunch buffet, the obese adolescents with BE consumed more total calories (+357.2 kcal), protein (+10.9 g), carbohydrates (+37.00 g), and fat (+18.46 g), albeit not statistically significant (Table 4).

We did not observe any significant correlations between energy/macronutrient intake with appetite sensations, appetite signaling proteins, glucose, or TFEQ categories. Similarly, appetite sensations (hunger and satiety) were not significantly correlated with appetite signaling proteins, glucose, or TFEQ categories in this cohort.

## 4. Discussion

We aimed to examine potential differences in appetite sensations, appetite signaling proteins, and glucose and their relationship with energy and macronutrient intake in obese adolescents with and without BE. There were no differences in hunger and satiety between obese adolescents with and without BE, albeit there was a nonsignificant trend towards impaired satiety in the BE group. Mirch et al. found no significant differences in the ratings of appetite sensations (hunger and satiety) in overweight children with and without BE prior to access to an* ad libitum* lunch following a standardized breakfast [10]. However, they found that children with BE had a shorter time spent satiated following a standardized breakfast with higher energy intake at the* ad libitum* lunch buffet [10]. This conflicts with Tanofsky-Kraff et al. who found that adolescents with LOC, regardless of BMI, did not have a higher energy intake at normal or binging meals as compared to the control group without LOC, with the exception of overweight and obese girls with LOC who had a higher energy intake during their binge meal [29]. Therefore, obese adolescents with BE may be hungrier or less satiated later in the study protocol for a given caloric intake even if they demonstrate similar initial hunger and satiety which may lead to overconsumption at a subsequent meal.

Obese adolescents with BE had significantly higher emotional eating scores when compared to the control group. This highlights the psychological aspect of the eating disorder in which negative emotions may be associated with eating as assessed by TFEQ questions such as “when I feel anxious, I find myself eating” or “when I feel blue, I often overeat”. Obese adolescence (10–16 years of age) with LOC had significantly more emotional and external eating behaviours as compared to a control group with no LOC [30]. There may also be a potential difference between these groups in uncontrolled eating as is evidenced by higher disinhibition scores in the obese individuals with BE, nonsignificant differences between the groups but a very large effect size (*d* = 1.056). This aligns with previous research demonstrating that overweight children with BE have a higher disinhibition score as compared to a control group [10]. Thus, obese adolescents with BE may exhibit a greater LOC or emotional eating at meal time which could potentially lead to increased energy intake over the course of the day.

In contrast to our initial expectations, postprandial response analyses showed no significant difference in ghrelin over time or between the two groups. Our results are in line with Monteleone et al. but contrast with Geliebter et al. [13, 14]. There were no temporal or group differences in PYY consistent with Geliebter et al. [19] but unexpected as it was originally postulated that there would be a smaller increase in PYY in obese adolescents with BE. As expected, postprandial response analyses showed a significant difference in glucose levels over time but with no difference between the two groups which is consistent with Geliebter et al. [14].

We acknowledge several limitations to our study. Given that many of the effect sizes obtained were moderate to large, an increased sample size may have provided the power to detect statistically significant differences and minimized the chance of type II error [31]. Moreover, using additional endocrine markers such as leptin, glucagon-like peptide-1, or cholecystokinin could provide a more comprehensive picture of appetite regulation in obese adolescents with BE. The fixed macronutrient breakfast was selected to study its effects on secretion of endocrine signaling proteins between the two different groups and was given to all subjects regardless of differences in body composition. As secretion is related to the quantity of the ingested nutrients, initial adjustment for body composition was not performed to prevent confounding of this effect. It has also been shown that obese individuals with full BED have a larger stomach capacity while obese individuals with subclinical forms of BED tend to have stomach capacities similar to nonbinging obese individuals [32]. Differences in stomach capacity may affect energy intake as Geliebter et al. has previously shown that stomach capacity is correlated with test meal intake [33]. It may also affect concentrations of signaling proteins as increased gastric capacity in obese individuals with full-fledged BED resulted in decreased fasting ghrelin levels [32]. The lack of significant differences in signaling proteins between the two groups may be somewhat influenced by the subjectivity of self-reported diagnoses and the subject's potential anatomical similarities to the control group. Therefore, a continuum scale of obese adolescents with full-fledged BED and subclinical BED as compared to a control group is needed to fully understand the spectrum of disordered eating behaviours associated with binge eating disorder [34]. Additional research has also proposed investigating LOC in children (6–12 years of age) as opposed to BE or BED due to the efficacy of LOC in identifying disordered eating in children [35].

## 5. Conclusions

Appetite sensations (hunger and satiety) did not differ between obese adolescents with and without BE. Lack of differences in appetite signaling proteins and glucose suggests that this may not be the best mechanism to investigate regarding etiology of the disorder. A significant difference between the two groups in terms of their emotional eating TFEQ cues highlights the importance that psychological factors play in relation to eating behaviour. This relevant finding could be used as a target in clinical practice to ensure control of these internal cues in obese adolescents with BE. The release of the new* Diagnostic and Statistical Manual for Mental Disorders: Fifth Edition* saw the inclusion of BED as an accepted eating disorder with a refined definition as it was previously only listed as a potential diagnostic category requiring further investigation. Overall, this highlights the importance of subclinical and full-fledged BED and need for further investigation regarding the psychological and metabolic effects of the binging and LOC episodes on obese adolescents.

## Figures and Tables

**Table 1:** Participant characteristics of the study population.

	Obese adolescents without BE (*n* = 9)	Obese adolescents with BE (*n* = 6)
Age (years)	13.7 ± 0.7	14.5 ± 0.8
Sex	6 M/3 F	1 M/5 F
Weight (kg)	99.8 ± 7.7	104.3 ± 6.8
Height (cm)	165.6 ± 3.0	164.7 ± 3.5
BMI (kg/m^2^)	35.9 ± 2.2	38.5 ± 3.0
Waist circumference (cm)	112.9 ± 3.6	118.0 ± 5.2
Fat mass (kg)	41.9 ± 7.0	53.7 ± 7.8
Fat %	41.8 ± 3.8	50.8 ± 4.2
Fasting VAS hunger^a^ (mm)	65.6 ± 12.1	87.2 ± 12.1
Fasting VAS full^a^ (mm)	30.5 ± 11.6	28.2 ± 11.6
Fasting ghrelin^b^ (pg/mL)	178.2 ± 20.3	202.0 ± 22.2
Fasting PYY^b^ (pg/mL)	74.9 ± 16.0	104.6 ± 17.5
Fasting glucose^b^ (mmol/L)	4.9 ± 0.3	4.9 ± 0.3

BE: subclinical binge eating disorder; BMI: body mass index; VAS: visual analog scale; PYY: peptide YY.

Effect size: fasting hunger (*d* = 0.799), fasting satiety (*d* = 0.089), ghrelin (*d* = 0.479), and PYY (*d* = 0.758).

^a^ *n* = 6 (non-BE) after adjusting for sex and body fat mass.

^b^ *n* = 7 (non-BE) after adjusting for sex and body fat mass.

**Table 2:** Measures of appetite sensations over the course of the entire study protocol (15–240 minutes) prior to the *ad libitum* lunch buffet, using 4 hr VAS AUC (mm × min).

	Obese adolescents without BE (*n* = 7)	Obese adolescents with BE (*n* = 6)
Hunger	17079.64 ± 1804.19	21096.26 ± 1969.96
Satiety	13693.39 ± 2049.18	7983.124 ± 2237.46

BE: subclinical binge eating disorder; VAS: visual analog scale; AUC: area under the curve.

Effect size: hunger (*d* = 0.910); satiety (*d* = 1.139).

**Table 3:** Three-Factor Eating Questionnaire responses after the standardized breakfast at 240 minutes postprandially.

	Obese adolescents without BE (*n* = 7)	Obese adolescents with BE (*n* = 6)	*P* value
Restraint^a^	12.33 ± 1.11	13.28 ± 1.21	0.60
Uncontrolled Eating^b^	17.68 ± 2.46	24.04 ± 2.69	0.14
Emotional eating^c^	5.07 ± 0.60	9.09 ± 0.65	0.002*

BE: subclinical binge eating disorder; ^a^range of score (6–24); ^b^range of score (9–36); ^c^range of score (3–12).

Effect size: restraint (*d* = 0.350); uncontrolled eating (*d* = 1.056); emotional eating (*d* = 2.750).

*Significant difference at *P* < 0.05.

**Table 4:** Nutrient intake profile at the “*ad libitum*” lunch buffet.

	Obese adolescents without BE (*n* = 7)	Obese adolescents with BE (*n* = 6)
Calories (kcal)	975.50 ± 164.7	1332.7 ± 179.8
Protein (g)	32.4 ± 4.6	42.4 ± 4.9
Protein (%)	13.2 ± 1.8	13.6 ± 2.0
Carbohydrates (g)	116.8 ± 19.8	153.8 ± 21.6
Carbohydrates (%)	47.1 ± 2.9	44.1 ± 3.2
Fat (g)	46.0 ± 9.6	64.5 ± 10.5
Fat (%)	39.6 ± 2.6	42.3 ± 2.9

BE: subclinical binge eating disorder.

Effect size: calories (kcal) (*d* = 0.862); protein (g) (*d* = 0.899); carbohydrates (g) (*d* = 0.764).
